# Aceruloplasminemia: A Severe Neurodegenerative Disorder Deserving an Early Diagnosis

**DOI:** 10.3389/fnins.2019.00325

**Published:** 2019-04-05

**Authors:** Giacomo Marchi, Fabiana Busti, Acaynne Lira Zidanes, Annalisa Castagna, Domenico Girelli

**Affiliations:** Department of Medicine, University of Verona, Verona, Italy

**Keywords:** iron metabolism, iron overload disease, neurodegeneration with brain iron accumulation, rare anemias, Aceruloplasminemia

## Abstract

Aceruloplasminemia (ACP) is a rare, adult-onset, autosomal recessive disorder, characterized by systemic iron overload due to mutations in the Ceruloplasmin gene (*CP*), which in turn lead to absence or strong reduction of CP activity. CP is a ferroxidase that plays a key role in iron export from various cells, especially in the brain, where it maintains the appropriate iron homeostasis with neuroprotective effects. Brain iron accumulation makes ACP unique among systemic iron overload syndromes, e.g., various types of genetic hemochromatosis. The main clinical features of fully expressed ACP include diabetes, retinopathy, liver disease, and progressive neurological symptoms reflecting iron deposition in target organs. However, biochemical signs of the disease, namely a mild anemia mimicking iron deficiency anemia because of microcytosis and low transferrin saturation, but with “paradoxical” hyperferritinemia, usually precedes the onset of clinical symptoms of many years and sometimes decades. Prompt diagnosis and therapy are crucial to prevent neurological complications of the disease, as they are usually irreversible once established. In this mini-review we discuss some major issues about this rare disorder, pointing out the early clues to the right diagnosis, instrumental to reduce significant disability burden of affected patients.

## Introduction

With the increasing awareness of an abnormal local iron accumulation in highly impacting neurodegenerative diseases such as Alzheimer’s and Parkinson’s disease (for review see references Ke and Ming, 2003; Ward et al., 2014), attention in understanding the peculiarities of iron metabolism in the brain has steadily raised (Raha et al., 2013; Rouault, 2013). CP is a copper-containing ferroxidase enzyme that plays a key role in cellular iron export, and has been postulated to have a neuroprotective function (Wang and Wang, 2018). In this context Aceruloplasminemia (ACP) represents a paradigmatic disorder highlighting how the loss of CP function causes iron accumulation and neurodegeneration.

Aceruloplasminemia is a rare, adult-onset, autosomal recessive disease caused by mutations in the *CP* gene, encoding CP. The impairment of CP ferroxidase activity results in pathological cellular iron retention and iron-mediated oxidative damage. The spectrum of clinical manifestations includes mild microcytic anemia, diabetes mellitus, liver disease, retinopathy, and progressive neurodegeneration due to iron accumulation in the brain and other parenchymal organs (Miyajima and Hosoi, 1993–2018; Kono, 2013). ACP is variably classified into different subgroups of rare diseases, such as neurodegeneration with brain iron accumulation (NBIA) (Hogarth, 2015), atypical microcytic anemias (Camaschella, 2013; Donker et al., 2014; Brissot et al., 2018), and non-*HFE* iron overload syndromes (Pietrangelo et al., 2011), depending on the specialists who see the patients for the first time. Apart from few neurologists and hematologists, the awareness of this disease is poor even among other specialists that could intercept the patients at various stages of their clinical history, such as diabetologists and ophthalmologists. Indeed, the commonly reported consistent diagnostic delay illustrates how ACP is under-recognized in most cases (Miyajima and Hosoi, 1993–2018; Vroegindeweij et al., 2015). This represents a major problem, since prompt diagnosis, and treatment are crucial to prevent neurological complications of the disease, which are usually irreversible once established. In this mini-review we outline some major issues about this rare disease, including the limited knowledge about epidemiology and genotype-phenotype correlation, as well as the challenges in diagnosis and therapy.

## Epidemiology

Our knowledge on the epidemiology of ACP is largely based on studies in the Japanese population, where the disease was first reported in 1987 by Miyajima and collaborators, through the description of a 52-year-old woman presenting with blepharospasm, retinal degeneration and diabetes mellitus (Miyajima et al., 1987). The same Authors later estimated the prevalence of the disease as being roughly 1:2,000,000 in Japanese individuals born from non-consanguineous marriages. Such estimation was inferred by a study in an adult population where serum CP was systematically measured with subsequent sequencing of the *CP* gene in individuals having low serum values (Miyajima et al., 1999). This estimation has obvious regional limitations, and cannot be applied in non-Japanese population, where ACP prevalence is actually unknown. Few more than one hundred of patients have been described in the literature so far, with a worldwide geographical distribution (Miyajima and Hosoi, 1993–2018; Kono, 2013; Doyle et al., 2015; Vroegindeweij et al., 2015; Pelucchi et al., 2018; Riboldi et al., 2018; Yamamura et al., 2018). While Japanese patients with neurological involvement still represent the majority (Miyajima and Hosoi, 1993–2018) the second group is represented by Italian patients reported by some referral centers for disorders of iron metabolism (Pelucchi et al., 2018). Nonetheless, ACP has been described also in other European countries (Spain, France, Netherlands, Belgium, Poland), as well as in China and in African American patients (Shang et al., 2006; Parks et al., 2013; Vroegindeweij et al., 2017; Riboldi et al., 2018). The generally low disease awareness, as well as a negative publication bias of single cases, represents possible explanations for the limited number of reports. An effort is needed to increase disease awareness especially among specialists in neurology, hematology, and endocrinology, in order to reduce the unacceptable diagnostic delay that is usually of decades after the appearance of the first biochemical and/or clinical signs of the disease (Miyajima and Hosoi, 1993–2018; Vroegindeweij et al., 2015; Pelucchi et al., 2018).

## Pathophysiology: Recent Advances and Unresolved Issues

**FIGURE 1:**
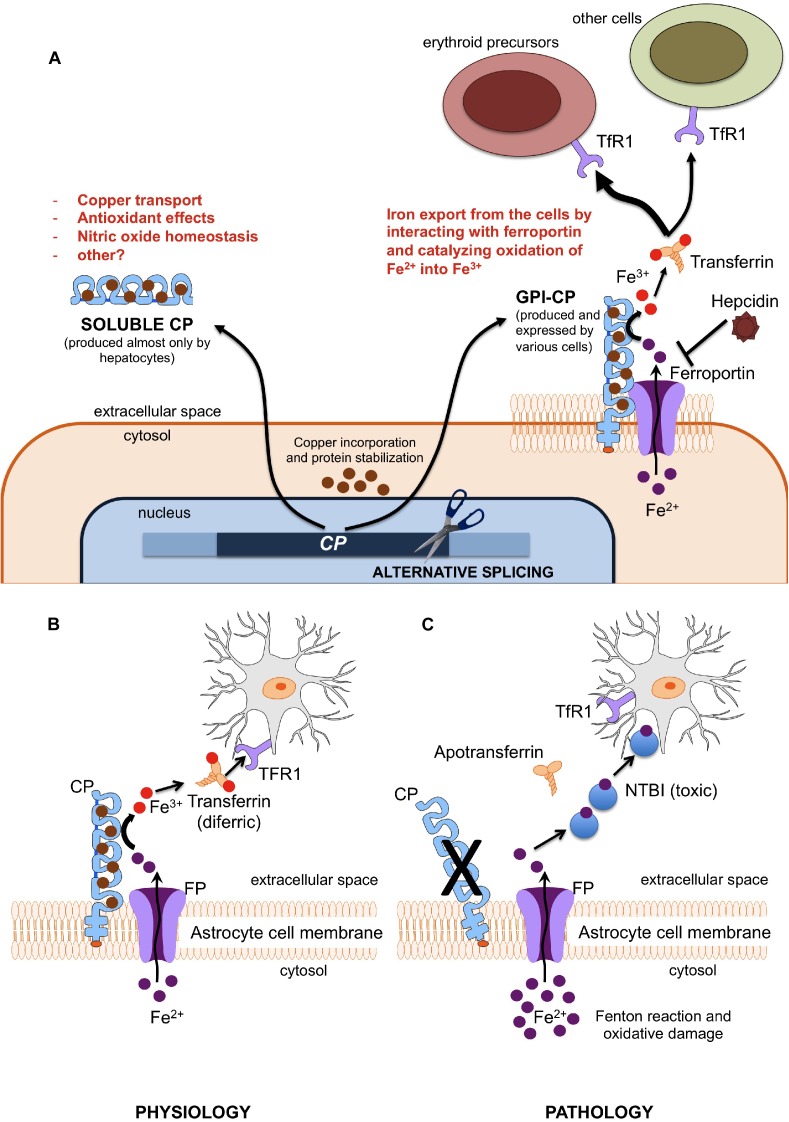
**(A)** Different functions of soluble and membrane-bound ceruloplasmin. Ferroxidase activity of membrane-bound CP is essential since only Fe^3+^ (but not Fe^2+^) can be incorporated into plasma transferrin, and then delivered to other cells (mainly erythroid precursors) via Transferrin Receptor 1 (TfR1). **(B,C)** Model of iron cross-talk between astrocytes and neuronal cells. **(B)** Ceruloplasmin is needed to oxidize Fe^2+^ into Fe^3+^, the only iron form that can be bound to transferrin for appropriate iron uptake by neurons through the transferrin receptor type 1 (TfR1). **(C)** Lack of ceruloplasmin leads to initial iron accumulation into astrocytes and starvation of neurons, with ensuing alteration of energetic metabolism and synthesis of neurotransmitters. This later stimulates the neuronal uptake alternative sources of iron, like non-transferrin-bound iron (NTBI), which is further toxic.

Aceruloplasminemia is caused by biallelic mutations in the *CP* gene, a 20 exons gene encompassing approximately 65 kb of DNA, located in chromosome locus 3q24-q25 and encoding CP (Yoshida et al., 1995; Hellman and Gitlin, 2002; Kono, 2013). CP is a single polypeptide chain of 1,046 amino acids that can bind up to six atoms of copper. The crystallographic 3D structure of the protein shows its six structural domains with a three-copper catalytic center that is crucial for its oxidative function (Bento et al., 2007). Copper is incorporated into CP before its secretion and this phase is crucial for its function and stability (Sato and Gitlin, 1991). Indeed, secretion of a copper-deficient CP leads to its rapid degradation in the plasma (Hellman and Gitlin, 2002). Two distinct isoforms of CP are produced by an alternative splicing in exons 19 and 20: a soluble form that is present in the plasma, and a glycosylphosphatidylinositol (GPI)-anchored membrane form (Patel et al., 2000). The soluble isoform is almost exclusively synthetized by hepatocytes, and accounts for near 95% of copper in the plasma (Hellman and Gitlin, 2002). On the other hand, the GPI-anchored membrane isoform is produced and expressed by a number of cells, including brain astrocytes glial cells, hepatocytes, macrophages, pancreatic, and retinal epithelial cells (Kono et al., 2010). The soluble form is involved in NO oxidation and homeostasis (Shiva et al., 2006), while the membrane isoform plays a key role in cellular iron egress (Muckenthaler et al., 2017). Indeed, CP cooperates with ferroportin, the ubiquitous and unique transmembrane protein able to export ferrous iron (Fe^2+^) from the cells (Drakesmith et al., 2015). This process needs to be completed by copper-mediated oxidation to ferric iron (Fe^3+^), to ensure the appropriate binding of extracellular iron to transferrin (Miyajima and Hosoi, 1993–2018). GPI-anchored CP has been also reported to interfere with the modulation of ferroportin activity by hepcidin (Nemeth et al., 2004; Kono, 2013; Aschemeyer et al., 2018), the master regulator of systemic iron homeostasis (Ganz, 2013). Membrane bound CP is therefore essential for ensuring a regular iron handling by various cells, including neurons that need iron for synthesis of neurotransmitters, energetic metabolism, and myelin formation (Miyajima and Hosoi, 1993–2018). According to the current model mainly based on animal experiments (Jeong and David, 2006), early neuronal injury in ACP may be related to the inability to uptake iron from the CP-deficient astrocytes. In advanced ACP, neurodegeneration may be due to iron accumulation and ensuing oxidative damage (Miyajima and Hosoi, 1993–2018; Kono and Miyajima, 2006; Kono, 2013), astrocytes loss and neuronal uptake of alternative and toxic iron sources, i.e., non-transferrin bound iron (NTBI) (Breuer et al., 2000). A basic model of astrocyte/neuronal iron handling is depicted in Figure 1. Lack of CP impairs this model, and, in general, ferroportin-mediated iron export from several cells. While this model explains iron maldistribution in critical cell types of ACP patients (e.g., astrocytes, neurons, hepatocytes, pancreatic, and retinal cells), it does not explain well why *total* iron stores are increased in such disease. This point needs the postulation of a ferroportin-mediated increase of dietary iron absorption by intestinal cells. Intestinal ferroportin, expressed on the basolateral membrane, works in synergy with the CP homolog hephaestin, another multicopper oxidase that is responsible for the transition Fe^2+^→Fe^3+^ needed for iron incorporation into plasma transferrin (Vulpe et al., 1999; Muckenthaler et al., 2017). Thus, this mechanism should be unaffected in ACP. Nevertheless, increased iron absorption should imply inappropriately low levels of hepcidin, the main negative regulator of ferroportin activity (Drakesmith et al., 2015; Muckenthaler et al., 2017). Indeed, low level of hepatic hepcidin expression has been reported in an ACP mouse model (Guo et al., 2009), and few ACP patients tested for serum hepcidin levels had levels in the lower range (Kaneko et al., 2010). While the latter finding needs confirmation in larger series, it remains to be explained why lack of CP leads to hepcidin suppression, particularly in patients with typically increased liver iron deposits that should stimulate rather than inhibit hepcidin production (Girelli et al., 2016). The iron-restricted erythropoiesis typically found in ACP (see below), due to impaired iron recycling, could stimulate the production of erythroferrone (ERFE) by bone marrow erythroid precursors, which in turn is a potent inhibitor of hepcidin (Kautz et al., 2014). However, up to now ERFE levels have never been measured in ACP patients. Anyway, things are likely more complicated than expected. For example, even if soluble CP is not able to pass the blood-brain barrier, it is also present in the brain through secretion by epithelial cells of the choroid plexus into the cerebrospinal fluid (Hellman and Gitlin, 2002). Moreover, the role of hephaestin may not be limited to intestinal iron absorption, since HEPH-KO mice have shown a dysregulation of brain iron homeostasis (Jiang et al., 2015).

Overall, it emerges that CP is part of a complex regulatory system that protects brain from either systemic iron deficiency or oxidative damage due to iron overload. In ACP the loss of this regulation results in neurodegeneration, as well as increased iron stores and toxic damage localized in other organs, especially liver, pancreas, and retina. A better understanding of the ACP pathophysiology could result in more efficient treatments, which for the moment are essentially limited to poorly effective iron chelation (see below).

## Genotype-Phenotype Correlations

Disease-related mutations in the *CP* gene are usually private, leading to absent or severely reduced ferroxidase activity of CP. To the best of our knowledge 28 missense, 17 frameshift, 13 splicing and 8 nonsense mutations have been described (Miyajima and Hosoi, 1993–2018; Pelucchi et al., 2018). Missense mutations lead usually to impairment of protein stability or alteration in copper binding domains. The majority of cases are due to homozygous mutations (which should prompt to ask for history of consanguinity), but compound heterozygosity has been also described. A milder disease course has been reported in some individuals with a single CP mutation (simple heterozygosity) (?. Possible explanations for such cases could be a second mutation in CP non-studied regions, in other relevant genes involved in genetic iron overload (compound heterozygosity) (Camaschella, 2005), or a dominant-negative effect of mutant CP through silencing the wild-type CP, i.e., causing retention also of the wild type CP in the endoplasmic reticulum (Kono, 2013). While no clear genotype-phenotype correlation is usually reported, recent observations suggest that some CP genotypes (i.e., homozygosity for Cys338Ser or for Ile991Thr) could be associated to residual ferroxidase activity, resulting in no or less frequent neurological impairment (Pelucchi et al., 2018). On the other hand, homozygosity for the Gly631Arg mutation has been associated in almost all cases to extra-pyramidal symptoms in Caucasian patients (Vroegindeweij et al., 2017).

## Clinical Features

Aceruloplasminemia is the only known iron overload disorder in which brain and systemic iron overload are combined. Neurodegeneration typically involves the dentate nuclei of cerebellum, basal ganglia, and thalamus, and is detectable by MRI showing in these regions diffuse hypointensity in T2^∗^ and T2 fast spin echo (FSE) sequences (McNeill et al., 2008a). The classical clinical triad of retinal degeneration, dementia and diabetes is frequently cited, mainly based on observations in Japanese patients (Miyajima et al., 2003). The largest review of clinical manifestations was based on 71 cases, most them being Japanese (Miyajima and Hosoi, 1993–2018). However, recent non-Japanese case series have highlighted a broader clinical and molecular heterogeneity (Vroegindeweij et al., 2017; Pelucchi et al., 2018). According to these descriptions, the clinical presentation of ACP usually leading to the diagnosis by neurologists includes cerebellar signs (dysarthria, ataxia of trunk, and limbs) and involuntary movements (dystonia, chorea, tremors), with an onset between 40 and 60 years of age (Miyajima and Hosoi, 1993–2018). At variance with this “typical” picture in Japanese ACP patients, cognitive-psychiatric changes and extrapyramidal signs are apparently more frequent in Caucasians, with a trend toward an earlier onset (Vroegindeweij et al., 2017). However, cognitive alterations (apathy, memory loss) and behavioral changes have a low specificity and can be often underestimated. When ACP is suspected on the basis of neurological symptoms, a brain MRI with specific sequences must be performed (McNeill et al., 2008a). In such cases, the differential diagnosis mainly includes other NBIAs and Wilson’s disease.

On the other hand, increasing reports point out the presence of a mild microcytic anemia as the earliest biochemical sign of ACP, in both Japanese and non-Japanese cases, which can be often traceable in patient’s history since childhood (Miyajima and Hosoi, 1993–2018; Pelucchi et al., 2018). Unfortunately, it rarely leads to the diagnosis of ACP in the early pre-symptomatic stage. Diabetes mellitus (DM) is another classical manifestation of ACP. It generally presents in the fourth to sixth decade, often in subjects without classical risk factors for diabetes, and requires insulin treatment (Vroegindeweij et al., 2017). As for mild anemia, in most cases of ACP DM is not appropriately recognized as part of a systemic disorder. The mechanism leading to DM in ACP is poorly understood. Of note, iron accumulation appears prevalent in exocrine rather than endocrine pancreas cells (Kato et al., 1997). Liver iron accumulation, reflecting systemic iron overload, is often present in ACP but rarely leads to clinically overt manifestations such as cirrhosis and liver failure (Miyajima and Hosoi, 1993–2018; Pelucchi et al., 2018). When liver biopsy is performed, iron accumulation is prevalent in hepatocytes like in classical *HFE*-related hemochromatosis, which can also be misleadingly considered in the differential diagnosis (Hellman et al., 2000). Retinopathy is frequently reported in Japanese ACP patients, but a uniform morphological description is lacking and it infrequently causes clinically relevant visual impairment (Miyajima and Hosoi, 1993–2018; He et al., 2007). In non-Japanese case series, retinopathy is far less frequent, and its attribution to ACP *per se* is often uncertain (Pelucchi et al., 2018). Finally, iron overload in other organs, such as heart and endocrine glands other than pancreas, has been sporadically reported in both Caucasian and Japanese patients, but the true prevalence may be actually under-investigated (Miyajima and Hosoi, 1993–2018; Badat et al., 2015).

The lack of uniform description of ACP cases makes it difficult to draw firm conclusions about the prognosis in these patients. Nonetheless, it is clear that neurological manifestations, when present, have the main impact on the quality of life of the patients and their families.

**FIGURE 2:**
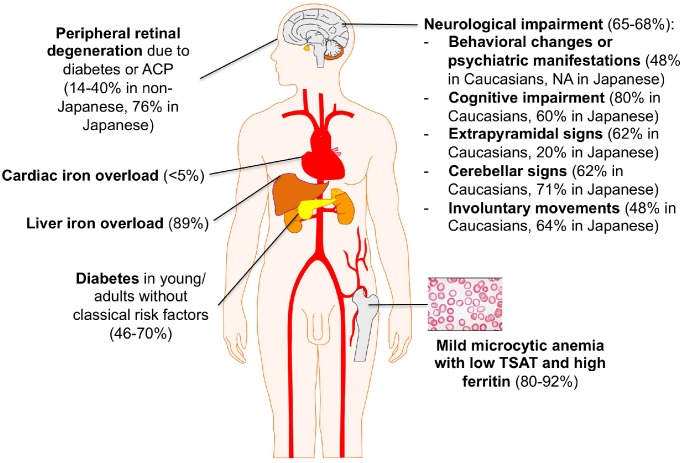
Clinical clues for ACP diagnosis. Percentages are taken from references (Miyajima and Hosoi, 1993–2018; Vroegindeweij et al., 2017; Pelucchi et al., 2018).

A summary of ACP main clinical features is depicted in Figure 2.

## ACP Diagnosis: Increasing Diagnostic Sensitivity by Using the “Biochemical” Triad Rather Than the “Clinical” Triad

As mentioned above, mild microcytic anemia, low transferrin saturation (TSAT), and hyperferritinemia are the first detectable signs of ACP according to all the major case series (Miyajima and Hosoi, 1993–2018; Pelucchi et al., 2018). This biochemical triad is pivotal in the definition of an “atypical microcytic anemia,” as opposed to typical cases due to iron deficiency in which serum ferritin levels are consistently low. Such triad may still be due to relatively frequent conditions, i.e., thalassemia syndromes and the so called anemia of inflammation, but once these hypotheses are easily discarded (i.e., by other obvious clinical clues and/or biochemical markers of inflammation), the differential diagnosis should consider a definite number of rare disorders. Besides ACP, they include ferroportin disease, congenital sideroblastic anemias, DMT1-deficiency and atransferrinemia (for extensive reviews see Camaschella, 2013; Donker et al., 2014; Brissot et al., 2018). In ACP, microcytic anemia and low TSAT are likely due to an impairment of iron recycling leading to an iron-restricted erythropoiesis. The resulting anemia is generally mild likely because other oxidation systems independent from CP can counteract the lack of this protein, allowing a certain degree of availability of iron for erythroid precursors. Anyway, in this condition the diagnosis of ACP long before other clinical manifestations can be easily done by assessing serum CP levels, which are usually undetectable or clearly below the normal range (Kono, 2013). Low CP levels can be detected also in asymptomatic *CP* heterozygotes, Wilson disease, Menkes disease or hypoproteinemias, which can be distinguished from ACP because of different clinical features before eventually molecular analysis. The majority of CP pathogenic mutations produce a dysfunctional and unstable apo- CP that is rapidly degraded in the plasma resulting in undetectable levels by conventional assays. However, in a minority of cases, serum CP can be only mildly decreased or normal. In such ultra-rare cases, a functional assay that explores the CP ferroxidase activity can be useful (Erel, 1998). This assay has also been reported of prognostic value, since residual ferroxidase activity has been associated to better neurological outcome, although in few observations (Pelucchi et al., 2018). Finally, low hepcidin levels are usually found in ACP patients, helping in the differential diagnosis of iron overload disorders (Kaneko et al., 2010; Girelli et al., 2016).

## Treatment of ACP: Still Unsatisfactory

Up to now, our knowledge on ACP treatment is mainly based on case reports. The most common approach is based on iron-chelating agents. At variance with deferoxamine and deferasirox, deferiprone is the only available iron chelator that is able to cross the blood-brain barrier, due to its low molecular weight and lipophilic properties (Kono, 2013). In general, all these drugs have shown encouraging results in reducing serum ferritin and iron accumulation in the liver (Finkenstedt et al., 2010; Kono, 2013). However, their efficacy on brain iron accumulation and neurological manifestations is controversial. Some studies indicate that they may prevent or slow down neurodegeneration, therefore early initiation of treatment is crucial (Pelucchi et al., 2018). However, the overall efficacy of iron chelation therapy in neurodegenerative disorders with brain iron accumulation is difficult to assess because of the lack of standardized description of clinical and MRI findings in ACP patients. According to a recent review, “there is no compelling evidence of the clinical effect of iron removal therapy on any neurological disorder,” including ACP (Dusek et al., 2016). Moreover poor tolerance to iron chelators, even including worsening of anemia due to further iron subtraction for erythropoiesis, has been often reported, limiting the long-term use of such agents that would be required to mobilize iron from the brain (Miyajima and Hosoi, 1993–2018; Mariani et al., 2004; Pelucchi et al., 2018). The efficacy of phlebotomies appears even lower than iron chelation, since CP deficiency likely impairs mobilization of tissue iron stores notwithstanding the induction of marked erythropoietic demand by blood removal. Indeed, failure to reduce liver iron overload, as well as development of neurological symptoms, have been occasionally reported in ACP patients treated with phlebotomies (Hellman et al., 2000; Watanabe et al., 2018). In few cases, iron chelation has been combined with fresh frozen plasma (FFP) administration (aimed to restore CP levels), with transient beneficial effects in a couple of studies (Yonekawa et al., 1999; Poli et al., 2017). Other strategies are based on preventing oxidative tissue damage by administration of vitamin E or zinc sulfate (Pelucchi et al., 2018). The latter has been associated to a dramatic neurological improvement in a patient with extrapyramidal signs and cerebellar movement disorder (Kuhn et al., 2007). Its effects on iron absorption, as well as the good tolerability make this drug a feasible option, also associated to iron chelation therapy (Donangelo et al., 2002). Tetracyclines have *in vitro* iron-chelating properties and are known to be able to cross the blood-brain barrier (Grenier et al., 2000). Amelioration of neurological dysfunction has been reported in a patient treated with minocycline (Hayashida et al., 2016). Finally, the administration of human CP to CP-knock-out mice was able to pass the blood-brain barrier, rescued brain ferroxidase activity, reduced neuronal death and brain iron deposits, and also ameliorated motor incoordination (Zanardi et al., 2018). Whether or not the use of a recombinant CP treatment could be successful in human ACP remains to be demonstrated.

## Concluding Remarks

Aceruloplasminemia is a rare proteiform disorder that can be faced by different specialists at different times. A high degree of suspicion is needed, and a proper early diagnosis is critical. To this end, the biochemical triad of mild anemia with low TSAT and high ferritin levels not due to any obvious alternative explanation is likely the best clue. The disease should be always suspected in any case of unexplained liver iron overload, diabetes mellitus in young adults with no classical risk factors, as well as in adult onset neurological dysfunctions (behavioral changes, psychiatric disorders, extrapyramidal or cerebellar signs) with MRI showing hypointensity in T2 FSE and T2^∗^ sequences in dentate nucleus of cerebellum, basal ganglia and thalamus. A better understanding of ACP molecular pathophysiology is needed, possibly leading to novel treatments in alternative to poorly effective current options. Considering the rarity of ACP and the lack of uniformity in cases described, a multicenter international registry should be instrumental to improve knowledge about this highly invalidating iron metabolism disorder.

## Author Contributions

GM wrote the manuscript. FB, ALZ, and AC co-wrote the manuscript. DG critically revised and edited the manuscript.

## Conflict of Interest Statement

The authors declare that the research was conducted in the absence of any commercial or financial relationships that could be construed as a potential conflict of interest.
